# Can We Use Neurocognition to Predict Repetition of Self-Harm, and Why Might This Be Clinically Useful? A Perspective

**DOI:** 10.3389/fpsyt.2016.00007

**Published:** 2016-01-27

**Authors:** Angharad N. de Cates, Matthew R. Broome

**Affiliations:** ^1^Unit of Mental Health and Wellbeing, Warwick Medical School, University of Warwick, Coventry, UK; ^2^Department of Psychiatry, University of Oxford, Oxford, UK; ^3^Warneford Hospital, Oxford Health NHS Foundation Trust, Oxford, UK

**Keywords:** self-harm, neurocognition, functional magnetic resonance imaging, structural magnetic resonance imaging, risk prediction, suicide, cognitive tasks

## Abstract

Over 800,000 people die by suicide each year globally, with non-fatal self-harm 20 times more common. With each episode of self-harm, the risks of future self-harm and suicide increase, as well as personal and healthcare costs. Therefore, early delineation of those at high risk of future self-harm is important. Historically, research has focused on clinical and demographic factors, but risk assessments based on these have low sensitivity to predict repetition. Various neurocognitive factors have been associated with self-harming behavior, but it is less certain if we can use these factors clinically (i) as risk markers to predict future self-harm and (ii) to become therapeutic targets for interventions. Recent systematic reviews and meta-analyses of behavioral tasks and fMRI studies point to an emerging hypothesis for neurocognition in self-harm: an underactive pre-frontal cortex is unable to respond appropriately to non-emotional stimuli, or inhibit a hyperactive emotionally-/threat-driven limbic system. However, there is almost no imaging data examining repetition of self-harm. Extrapolating from the non-repetition data, there may be several potential neurocognitive targets for interventions to prevent repeat self-harm: cognitive training; pharmacological regimes to promote non-emotional neurocognition; or other techniques, such as repetitive transcranial magnetic stimulation. Hence, there is an urgent need for imaging studies examining repetition and to test specific hypotheses. Until we investigate the functional neurocognitive basis underlying repetition of self-harm in a systematic manner using second-generational imaging techniques, we will be unable to inform third-generational imaging and potential future clinical applications.

## Self-Harm and Risk Assessment for Prediction of Its Repetition

### What Is Self-Harm and Why Is It Important?

Self-harm, where an individual intentionally causes physical harm to themselves by self-injury or self-poisoning irrespective of motivation (1, 2), affects both those with and without previously diagnosed mental illness. Any episode of self-harm potentially results in (i) serious morbidity or (ii) death by suicide. Studies suggest that between one in 20 and one in 40 self-harm episodes with reported intent to die ends in completed suicide (3–5). Globally, over 800,000 people die by suicide annually (6–8), and it is estimated that by 2020, suicide will contribute more than 2% to the global burden of disease (9). In the UK, and internationally, the total number of suicides in the general population has been rising since 2009 (8, 10, 11) with this rise most marked in men aged 45–54 years (10). However, self-harm and suicide remain important throughout the lifespan: suicide is the second most common cause of death in young people in the UK (12) with a conservative estimate of 10% of young people reporting at least one episode of self-harm (13).

Historically, the over-arching term “self-harm” has been divided into “parasuicide” (no reported intention from the individual concerned to die) and “attempted suicide” (where there is a reported intention to die). However, reported intent of previous episodes of self-harm does not appear to correlate with future self-harm or suicide (14–16). This may be because of conscious or subconscious underreporting by the individual, or it may be because the pathophysiology of self-harm and the potential future pathway is the same regardless of previous conscious intent. For this reason, both the World Health Organization (WHO) and the UK National Institute of Health and Care Excellence (NICE) include all behavior regardless of method or intent in their definitions of self-harm (1, 2). However, intent remains used as a delineating factor particularly in the US to separate non-suicidal self-injury (NSSI) from suicidal attempts, which are then treated separately for clinical and research purposes. However, recent research shows that self-harm method switching occurs routinely in individuals (17). If, in fact, this dichotomy is false, by separating out self-harm on presumed or purported intent or method used we may be reducing the number of available participants for studies into self-harm.

### How Have Predictors of Self-Harm Been Approached and Analyzed?

Prevention of suicide remains difficult, partly because the complex underlying cognitive processes remain only partially understood (18, 19). The few successful prevention strategies have appeared to focus on public health level interventions, such as paracetamol sale restrictions (20). Suicide prevention is in need of markers that predict future self-harm and serve as a basis for intervention (21). To date, self-harm research has focused on retrospective identification of high-risk individuals following self-harm using demographic and clinical factors, e.g., age, sex, diagnosis of mental illness (22–25). This is because this information is easily achievable from patient records rather than requiring direct patient contact (26, 27), which is more difficult, costly, and has many ethical implications. However, the increasing suicide rate of late despite our improved understanding of demographic and clinical factors indicates that these factors are inadequate in terms of predicting future risk of self-harm alone. Standard risk assessment tools based on these factors, such as the SADPERSONS tool, do not appear to accurately predict individuals requiring psychiatric admission or community aftercare, or to predict those who repeat self-harm (28). Furthermore, the 2014 UK confidential inquiry report on suicides in primary care found that 37% of those who died by suicide between 2002 and 2011 did not have a mental health diagnosis recorded on their GP records (11). This reinforces the fact that to prevent suicides we need to learn how to identify high-risk people in those not previously known to mental health services or with known mental illness.

### What Is Already Known about Predicting Repetition of Self-Harm?

There is almost ubiquitous evidence that the most important predictor of future self-harm is past self-harm (14, 29–31). After a first episode of self-harm, approximately one in six patients repeats self-harm over the first year, and one in four after 4 years (14). With repetition, there is an increasing risk of suicide (32) and increased costs personally and to the health service (33). Therefore, the study of prediction of repetition of self-harm is very important to try and reduce the personal, clinical, social, and financial burdens of self-harm and suicide.

Repetition of self-harm has been associated with various demographic and clinical factors, including (i) sociodemographics [extremes of age, and low educational level (30, 34), being unmarried (35), and being unemployed (36)]; (ii) personal history [abuse in childhood (37)]; and (iii) specific mental disorder diagnosis [personality disorder (38), anxiety disorder (39), depression (27), and substance and alcohol misuse (40, 41)]. However, although up to 90% of people who die by suicide have a psychiatric disorder (42), making this a risk factor for self-harm, most people with a mental illness will not self-harm. Thus, psychiatric diagnosis is not very helpful in terms of predicting suicide or why an individual might do so (19). The most commonly used risk assessment tool in England based on clinical and demographic factors, the SADPERSONS tool (43), does not accurately predict individuals requiring psychiatric admission or community aftercare, or repeat self-harm (28).

### Why Is Repetition of Self-Harm Particularly Important?

In the longer term, it appears possible that repetition of self-harm in young people may act as a marker of an emerging wider psychopathological process, resulting in long-term contact with mental health services and need for care. The self-harm itself may or may not persist (44, 45), but young people who self-harm repeatedly appear at greater risk of serious mental illness and poor educational and occupational outcomes in later adulthood (45). Therefore, a greater understanding of repeat self-harm, and its neurobiological basis, is vital to be able to both predict and prevent suicide, but also to alleviate current and long-term mental distress, and to aid early detection of young people at high risk of future psychiatric difficulties.

## Neurobiological Basis for Neurocognition in Self-Harm

### Self-Harm as a Complex Clinical Syndrome at Least Partly Independent of, but Influenced by, Diagnosis

Prediction of future risk of self-harm is likely to be enhanced if this non-individualized data (demographic and clinical factors) could be combined with more personalized individual-based factors, such as personality and cognition. Self-harm is likely to be best understood as an interaction between underlying individual susceptibilities to self-harm (such as personality and cognitive factors) and current social and health stressors (such as employment or financial issues, mental and physical illness, and negative life events), known as a “stress–diathesis model” (18, 19, 46, 47). Various stress–diathesis models of self-harm exist, typically based on the concept that self-harm may represent a clinical syndrome in its own right (19, 47, 48). This is consistent with recent National Institute of Mental Health guidance in the US which has reported that it will only commission and fund future research if it crosses existing diagnostic boundaries and instead focuses on clinical syndromes (49). However, for example, Hawton et al. found that depression, in particular, was a consistent predictor of repetition (27), indicating the importance of appreciating stress–diathesis models within the context of potential particular psychiatric diagnoses (as potential and common stressors), both in terms of risk prediction and also for future management planning.

### Current Evidence for Neurocognition in Self-Harm

Although self-harm behavior includes much heterogeneity, the underlying demographic, clinical, and neurobiological factors are likely to be similar across individuals (47). There are putative genetic and molecular markers for self-harm behavior involving abnormalities at the neurochemical and cellular level (47). There has also been exploration into regional brain structural abnormalities in patients who have a history of self-harm and deficits of the associated brain functions (known as neurocognitive correlates) (18, 21). These neurocognitive factors may act as objective markers of self-harming behavior, overcoming self-reporting biases (47, 50). Therefore, neuroimaging studies in self-harm have a vital role, allowing us to connect structural brain abnormalities with functional and cognitive changes, and thereby produce a connected neurobiological theory to suicidal behavior.

van Heeringen and colleagues have conducted several reviews of neuroimaging studies investigating self-harm behavior (21, 47, 51). Their most recent systematic review and meta-analysis of structural and functional MRI studies examined general suicidal behavior in those with a mental illness only (21). They identified activation foci from 12 studies including 475 participants for meta-analysis (213 suicide attempters with mental illness, 262 psychiatric controls), six of which examined structural findings only, and six functional findings only. A separate narrative review identified 21 studies of structural MRI and 9 studies of functional MRI performed in those with previous suicide attempts (47). Blumberg and colleagues also published a recent review into neurobiological risk factors identified for suicidal behavior using any form of neuroimaging (52), but again their review was limited to those studies involving suicidal behavior in the context of an underlying mental illness.

These reviews indicate that specific structural findings are associated with self-harm behavior, such as reduced gray matter in the orbitofrontal cortex (OFC), the dorsolateral pre-frontal cortex (DLPFC), the anterior cingulate cortex (ACC), the insula and superior temporal gyrus, and basal ganglia, and increased volume of the amygdala (21, 47, 51, 52). There is also evidence of white-matter hyperintensities, and increased inferior frontal white-matter tracks bilaterally (such as the uncinated fasciculus and the inferior orbital fasciculus), indicating deficits in the connections between these structural areas (47, 52). Diffusion tensor imaging (DTI) studies also support anterior white-matter abnormalities and suicidal behavior (52), with identified abnormalities in frontal cortex and basal ganglia white-matter connections. These structural and connective abnormalities point to potentially impaired functioning of the amygdala–orbitofrontal–cingulate network (53), which prevents the amygdala from inhibiting the OFC and PFC, and the OFC from inhibiting the ACC appropriately. Genetic factors may be involved in these changes in basic brain structure and circuitry. For example, pre-frontal and other brain volume abnormalities are seen in first-degree relatives of those with a history of suicidal behavior, as well as in the individuals themselves (54, 55).

Functional neuroimaging studies [fMRI, single photon emission computerized tomography (SPECT), positron emission tomography (PET)] and off-line neuropsychological studies detailed and referred to in these reviews and elsewhere have been used to investigate the neurocognitive correlates of these brain regions linked to self-harm.

(i) *Decision-making:* activation of the ACC, a key player in effort-based decision-making (56), is different in young people with a previous self-harm and depression compared to those with depression but no self-harm (57). Involvement of the ACC in the process of self-harm may also explain findings of poorer Stroop performances off-line and during imaging studies in those with previous self-harm (18, 21). The OFC also appears to be related to self-harm and to decision-making. However, involvement of the OFC may relate to decisions determining reward expectation and delay (58), including “risky decision-making.” Off-line, risky decision-making has been found in euthymic patients with suicidal behavior as well as healthy biological relatives of suicide completers, suggesting that it is an endophenotype with trait-like characteristics (46, 59, 60).
(ii) *Emotional-processing:* impaired processing of emotional feedback appears to be associated with self-harm behavior in both adults and adolescents in fMRI studies (61, 62). Aberrations in serotoninergic activity due to poor functioning of the PFC appear in patients with previous self-harm behavior (52), resulting in multiple deficits, including the ability to process emotional stimuli in a controlled manner (47). Furthermore, carriers of a particular (S) allele of the serotonin transporter gene, 5HTTLPR, appear to have reduced functional connectivity between the ACC and amygdala (63, 64). Thereby, emotional-processing and self-harm appear to be connected in terms of genetic, structural, connective, and functional studies.
(iii) *Memory:* impairments in memory also seem to be present in patients with self-harm with and without mood disorder (65), although there is little in terms of neuroimaging evidence. Off-line, working memory and executive function deficits (for example, on the Iowa gambling task and verbal fluency) in particular are associated with self-harm in the context of mood disorders (18). A recent systematic review of studies in psychiatric patients found that autobiographical memory was significantly less specific and more general in patients with a previous suicide attempt relative to those without, and long-term and working memory were both more impaired in suicide attempters than in patient and healthy controls (66).

In their recent synthesis and meta-analysis of the fMRI data relating to neurocognition and self-harm in mental illness (21), van Heeringen and colleagues found a cluster in the dorsal ACC showing increased activation in suicide attempters when compared to psychiatric controls during exposure to angry faces or mildly happy faces, while activation was reduced in suicide attempters versus psychiatric controls for high-risk decisions. Similarly, a cluster in the rostral ACC showed increased activation in suicide attempters compared to psychiatric controls during exposure to angry faces while activation was reduced in attempters compared to controls during Go/No-Go tasks. Therefore, synthesis of evidence from off-line studies, and structural and functional imaging, indicates the following hypothesis of neurocognition in self-harm: in those with self-harm, there appears to be increased activation during emotional tasks (such as exposure to emotionally charged faces) in the ACC, and decreased activation during non-emotional cognitive tasks (such as decision-making) in the ACC (21). In van Heeringen’s review, there were no studies found directly linking structural and functional changes in the brain in the context of self-harm (21). However, he suggests that his functional meta-analysis may be put in the context of the known structural deficits previously described above (21). In other words, an underactive pre-frontal cortex is unable to respond appropriately to non-emotional stimuli, or inhibit a hyperactive emotionally-/threat-driven limbic system.

### Current Evidence for Neurocognition in Repetition of Self-Harm, and Its Use in Prediction

Repetition of self-harm with an increasing risk of suicide can be understood as an “escalating disinhibition syndrome.” Therefore, studies examining repetition and facets of executive control (67) are likely to be very important, such as response inhibition, interference, attention, decision-making, and cognitive flexibility.

However, there is almost no imaging data examining repetition of self-harm. We recently conducted a systematic review (de Cates et al., in preparation) into repetition of self-harm and neurocognition, which indicated that there is only one published conference abstract of an imaging study examining emotional-processing (68). In terms of off-line studies, only a very few studies have examined decision-making (69–71), although each of these demonstrated evidence of an association between impaired decision-making and increased risk of repetition. There were also associations for repetition of self-harm with specific attentional biases on a modified emotional-Stroop test (72); specific results on the test predicted future self-harm better than underlying mood disorder or clinician ratings. Cognitive inflexibility predicted future suicidal ideation in those with a past history of self-harm (73). Rasmussen and colleagues found an association between recall of positive autobiographical memories, but not negative memories, and repetition of self-harm in an exploratory study (74). These studies indicate that poor functioning or impairments in terms of neurocognition may be associated with increased risk of repetition. However, it is less clear if we can apply these correlates clinically: that is, use assessment of these factors in an individual to predict the risk of future self-harm. This personalized neurocognitive profile might also provide potential targets for therapeutic interventions aimed at reducing this risk.

## How Could Neurocognition be Used to Guide Treatment for Repetition of Self-Harm?

Extrapolating from the non-repetition data, there may be several potential neurocognitive avenues to identify risk of repeating self-harm, and potential modes of intervention. Psychotherapy techniques may be helpful, such as cognitive therapy or training (75, 76), or dialectical behavioral therapy in situations such as personality disorder (77). However, there have been few replications of psychological therapies for self-harm (19) and no specific work in multiple attempters. For adolescents with multiple episodes of self-harm, mentalization therapy (based on understanding actions in terms of thoughts and feelings) (78) shows promise at reducing repetition frequency (79), but the effect was modest and the one trial was small (80). Non-invasive neurophysiological techniques, such as repetitive transcranial magnetic stimulation (rTMS), may prove to be effective (81–83). For example, we can increase risky decision-making in men by inhibiting cortical function with TMS (84). Therefore, may it be possible to improve cognition and ineffective decision-making by a similar method, for example, as van Heeringen suggests, by directing rTMS at the DLPFC to modify function in the OFC and hopefully reduce risky decisions (47)?

Previous investigations into potential medical treatments for self-harm have included antidepressants, mood stabilizers, and natural products, but no significant treatment effect on repetition of self-harm was found for any of these options in a recent Cochrane review, although the quality of evidence was low (85). Only one antipsychotic, Flupenthixol showed promise, but again the quality of the evidence was very low. However, perhaps we are not examining the correct potential medication in the correct circumstance. We know that emotional processing, in particular, is controlled by serotonin, and relates to amygdala functioning. However, amygdala responses to fearful faces are modified by antidepressant use in depressed patients (86, 87). If we could identify a group of individuals with self-harm behavior and emotional-processing deficiencies, might they receive a particular benefit from antidepressants? A large body of evidence indicates that Lithium and Clozapine are anti-suicidal unrelated to their efficacy as a mood stabilizer and antipsychotic, respectively, possibly related to their serotoninergic effects (88, 89), as well as Lithium’s effect of reducing cell death and potentially increasing brain volume (90). However, as yet, there has been little or no practice of using Lithium or Clozapine as a prevention strategy outside of mood disorder (or violent behavior), or psychotic disorder, respectively. These may also be an avenue for further exploration. Furthermore, could we consider existing pharmacological regimes than have evidence for promoting non-emotional neurocognition and memory in other disorders, such as acetylcholinesterase inhibitors typically used for Alzheimer’s dementia? Another possibility for further research is ketamine, an NMDA antagonist that is finding increasing support for its use as an antidepressant (91). Rapid suppression of suicidal ideation has also been noticed in depressed patients treated with ketamine (92), making it a potential treatment for high-risk suicidal ideation to prevent imminent self-harm (93). However, the specific brain regions involved in ketamine’s ability to reduce suicidal ideation are unknown and much further work is required, including animal studies or similar before it can be considered for clinical trials.

Therefore, the dearth of imaging studies examining repetition in particular is concerning considering the individual, financial, and social implications of people who repeat self-harm frequently and/or die by suicide. Until we investigate the functional neurocognitive basis underlying repetition of self-harm in a basic-science (i.e., brain regions and networks) and systematic manner using second-generational imaging techniques, we will be unable to inform third-generational imaging and potential future clinical applications, of which there may be many. Potentially combining research into imaging and genetics may yield fruit, particularly in terms of neurochemistry alleles. However, clearing several process issues may help, such as reaching a consensus in terms of phenomenology of self-harm and associated thoughts and behaviors.

## Author Contributions

Both authors devised the article topic. Both authors contributed to the original research detailed in the article. AdeC drafted the article and both authors revised the article.

## Conflict of Interest Statement

Both the authors declare that the research discussed in this manuscript was conducted in the absence of any commercial or financial relationships that could be construed as a potential conflict of interest.
